# YY1 accelerates oral squamous cell carcinoma progression through long non-coding RNA Kcnq1ot1/microRNA-506-3p/SYPL1 axis

**DOI:** 10.1186/s13048-022-01000-5

**Published:** 2022-07-01

**Authors:** Yi Ding, Heng Duan, Jian Lin, Xuanxuan Zhang

**Affiliations:** 1 Center for Drug Research and Development, Guangzhou Key Laboratory of Construction and Application of New Drug Screening Model Systems, Key Laboratory of New Drug Discovery and Evaluation of Ordinary Universities of Guangdong Province, Guangdong Pharmaceutical University, No. 280, Waihuan East Road, Guangzhou, 510006 Guangdong China; 2 School of Life Sciences and Biophamaceutics, Guangdong Pharmaceutical University, Guangzhou, 510006 Guangdong China; 3 Department of Pharmacy, Stomatological Hospital, Southern Medical University, Guangzhou, 510280 Guangdong China; 4 The Seventh Affiliated Hospital, Sun Yat-Sen University, Shenzhen, 518107 Guangdong China

**Keywords:** Oral squamous cell carcinoma, Ying Yang1, Long non-coding RNA KCNQ1 overlapping transcript 1, MicroRNA-506-3p, Synaptophysin like 1

## Abstract

**Objective:**

Ying Yang1 (YY1) has already been discussed in oral squamous cell carcinoma (OSCC), but the knowledge about its mediation on long non-coding RNA KCNQ1 overlapping transcript 1/microRNA-506-3p/synaptophysin like 1 (Kcnq1ot/miR-506-3p/SYPL1) axis in OSCC is still in its infancy. Hence, this article aims to explain the mechanism of YY1/Kcnq1ot1/miR-506-3p/SYPL1 axis in OSCC development.

**Methods:**

YY1, Kcnq1ot1, miR-506-3p and SYPL1 expression levels were determined in OSCC tissues. The potential relation among YY1, Kcnq1ot1, miR-506-3p and SYPL1 was explored. Cell progression was observed to figure out the actions of depleted YY1, Kcnq1ot1 and SYPL1 and restored miR-506-3p in OSCC. OSCC tumorigenic ability in mice was examined.

**Results:**

Elevated YY1, Kcnq1ot1 and SYPL1 and reduced miR-506-3p were manifested in OSCC. YY1 promoted Kcnq1ot1 transcription and up-regulated Kcnq1ot1 expression, thereby promoting OSCC cell procession. Silencing Kcnq1ot1 or elevating miR-506-3p delayed OSCC cell progression and silencing Kcnq1ot1 impeded tumorigenic ability of OSCC cells in mice. YY1-mediated Kcnq1ot1 sponged miR-506-3p to target SYPL1.

**Conclusion:**

YY1 promotes OSCC cell progression via up-regulating Kcnq1ot1 to sponge miR-506-3p to elevate SYPL1, guiding a novel way to treat OSCC.

**Supplementary Information:**

The online version contains supplementary material available at 10.1186/s13048-022-01000-5.

## Introduction

Oral squamous cell carcinoma (OSCC) arises de novo, accompanied by latent malignant lesions, including erythroplakia and leukoplakia [1]. In most cases of oral and maxillofacial cancers, OSCC stands with hostile and invasive properties [2]. Surgical resection, immunotherapy, chemotherapy and radiotherapy are inclusive in the standard treatments of OSCC [3]. Miserably, due to diagnosis in the late stage and local recurrence after surgical resection, OSCC patients often experience an unwanted survival [4]. The catastrophic disease has imposed great sufferings on patients, which gives an impetus to search for effective therapeutic agents.

Yin Yang 1 (YY1) has been investigated as a transcriptional factor which enhances esophageal squamous cell carcinoma (ESCC) cell proliferation [5]. Also, a liaison has been observed between overexpressed YY1 and inferior survival of tongue squamous cell carcinoma (TSCC) patients, indicating that YY1 may regulate the malignant phenotype of SCC [6]. LncRNA KCNQ1 overlapping transcript 1 (Kcnq1ot1) is the one, whose transcription can be activated by YY1 to up-regulate its own expression [7]. Kcnq1ot1 has been recently studied to induce cisplatin resistance for tongue cancer via regulation of microRNA (miR)-124-3p [8]. Moreover, Kcnq1ot1 is regarded as a promoter for tumorigenic activities of OSCC cells through modulating miR-185-5p [9], and an activator for enhancing TSCC cell proliferation and cisplatin resistance by mediating miR-211-5p [10]. The interaction network of lnRNA-miRNA is also proved between Kcnq1ot1-miR-506-3p [11]. miR-506-3p is defined as a modifier of proliferation and motility of ESCC cells [12]. miR-506, abnormally expressed in tissue samples, has the potential application in the diagnosis and prognosis of OSCC [13], and could block cellular metastasis in OSCC [14]. Synaptophysin like 1 (SYPL1) is a synaptic vesicle membrane protein that participates in cancer development such as colorectal cancer (CRC) and papillary thyroid carcinoma, and can serve as a biomarker for disease diagnosis [15, 16]. In general, YY1, Kcnq1ot1, miR-506-3p and SYPL1 have the applicable prospects in treating cancers, but the mechanism focused on their loop feedback in OSCC was rarely elaborated. Therefore, YY1/Kcnq1ot1/miR-506-3p/SYPL1 axis becomes the pivot of this study.

## Methods and materials

### Ethics statement

The study protocol was approved by the Ethics Committee of the Guangdong Pharmaceutical University. The written informed consent of all patients was obtained. Animal experiments were approved by the Animal Protection and Use Committee of the Guangdong Pharmaceutical University.

### Study design

This study was to investigate the effect of YY1-regulated Kcnq1ot1 on the growth and development of OSCC cells through the regulation of SYPL1 expression by miR-506-3p. Clinical samples were collected and subjected to RT-qPCR to detect the expression levels of YY1, Kcnq1ot1, miR-506-3p and SYPL1. Human oral keratinocytes (HOK) and OSCC cell lines were cultured, and the expression levels of YY1, miR-506-3p, Kcnq1ot1, and SYPL1 in each cell line were detected by RT-qPCR. Through the differential expression of genes in each OSCC cell line, two target cells in this study were screened for subsequent experiments. In both target cells, MTT assay and colony formation assay were utilized for detecting cell proliferation, flow cytometry for measuring cell apoptosis, Transwell assay for determining cell migration and invasion after interference with YY1, Kcnq1ot1, miR-506-3p, and SYPL1. An OSCC mouse model was established. After treatment, the tumor volume and weight of the mice were recorded, and the tissues were stained with TUNEL to elucidate the effect of Kcnq1ot1 on OSCC in vivo.

### Clinical specimen collection

From patients undergoing surgery, 98 pairs of OSCC tissues and normal tissues were obtained. None of them had received radiotherapy and chemotherapy before surgery.

### Cell culture

Human oral keratinocytes (HOK) and human OSCC cell lines (Cal-27, SCC-25, SCC-9, and HN4) were collected from the American Type Culture Collection (Manassas, USA). HOK were cultivated in a HOK culture medium (ScienCell, Carlsbad, CA, USA) whereas Cal-27, SCC-25 and HN4 cells in Dulbecco's modified Eagle medium (DMEM), and SCC-9 cells in DMEM/F12. Cells were protected against mycoplasma contamination. All of the media were supplemented with 10% fetal bovine serum (FBS; Hyclone, Israel) and 100 U/mL penicillin and streptomycin (Invitrogen, CA, USA) [17].

### Cell transfection

The coding sequence of YY1 was inserted into pcDNA3.1( +) to generate pc-YY1, with empty vector as a negative control (NC). siRNAs for YY1, Kcnq1ot1, SYPL1 and their corresponding NCs, along with miR-506-3p mimic and its NC were all obtained from GenePharma (Shanghai, China). Each transfectant was transfected into Cal-27 or SCC25 cells by Lipofectamine 3000 (Thermo Fisher Scientific, MA, USA). Successfully-transfected cells were divided into the following groups: si-NC, si-Kcnqlot1, Vector, pcYY1, si-YY1, pcYY1 + si-Kcnqlot1, mimic-NC, mimic-miR-506-3p, si-NC and si-SYPL1 groups [18].

### Reverse transcription quantitative polymerase chain reaction (RT-qPCR)

Total RNA of tissues and cells was obtained by Trizol (Invitrogen). PrimeScript RT Reagent Kit (TaKaRa, Dalian, China) and Mir-X™ miRNA First-Strand Synthesis Kit (TaKaRa) were utilized for reverse transcription of the extracted RNA into cDNA. The cDNA was then diluted and subjected to RT-qPCR using SYBR Green Master Mix (TaKaRa) by using a CFX Real-time PCR system (Bio-Rad, Hercules, CA, USA). The primers for PCR were listed in Supplementary Table 1. Taking glyceraldehyde-3-phosphate dehydrogenase and U6 as internal controls, the gene expression was calculated by 2^−ΔΔCt^ [19].

### Western blot assay

Total protein was extracted using radio-immunoprecipitation assay (RIPA) lysis buffer (Gibco, Carlsbad, CA, USA) containing phenylmethanesulfonyl fluoride (PMSF), and tested by a bicinchoninic acid kit (Sangon, Shanghai, China). Separated through sodium dodecyl sulphate polyacrylamide gel electrophoresis, the protein samples were electroblotted to polyvinylidene fluoride membranes and blocked with 5% skim milk. After that, membranes were probed with anti-YY1 (1:1000; Santa Cruz Biotechnology, CA, USA) and SYPL1 (1:1000; Abcam, MA, USA) overnight, and with the corresponding secondary antibody for 2 h. Protein bands were developed by enhanced chemiluminescence (Millipore, MA, USA).

### 3-(4, 5-dimethylthiazol-2-yl)-2, 5-diphenyltetrazolium bromide (MTT) assay

Cal-27 and SCC25 cells were cultivated in a culture medium on 96-well plates at 6 × 10^3^ cells/well. MTT buffer was added into each well at post 24 h, 48 h, and 96 h cultivation. Subsequently, cells were cultivated for another 6 h to measure optical density (OD_570 nm_) on a microplate reader (Thermo Fisher Scientific) [20].

### Colony formation assay

Cal-27 and SCC25 cells were cultured for 21 d in 10% FBS-DMEM on 6-well plates (Corning, NY, USA) at 200 cells/well. Colonies fixed with methanol and stained with 0.5% crystal violet (Sigma-Aldrich, MO, USA) were counted.

### Flow cytometry

Cell apoptosis rate was determined by eBioscience Annexin V-fluorescein isothiocyanate (FITC) Apoptosis Detection Kit (Thermo Scientific). Cal-27 or SCC25 cells after detachment were resuspended in 1 × binding buffer and reacted with propidium iodide and FITC-Annexin V. The cell apoptosis rate was evaluated on a flow cytometer (FACScan, BD Biosciences, Franklin Lakes, NJ, USA). 

### Transwell assay

Cell invasion and migration were examined by Transwell chamber (Corning) with or without matrigel (BD Biosciences). The upper side was filled with serum-free medium and added with cell suspension (200 μL) whereas the lower side contained 10% FBS-DMEM. After 24 h, with cells on the upper side wiped off, those on the lower side were fixed with 4% paraformaldehyde and stained with crystal violet buffer. Lastly, cells were photographed and quantified in 5 random fields [21].

### Chromatin immunoprecipitation (ChIP)

A commercially kit (Beyotime, Shanghai, China) was applied for ChIP. Cells were cross-linked with 1% formaldehyde and sonicated on ice to produce 200–500 bp fragments. The chromatin was probed with anti-YY1 or immunoglobulin G. The precipitated chromatin DNA was examined by RT-qPCR [22].

### RNA pull down assay

Pre-cooled cells were incubated with RNA probe labeled with high-affinity biotin and 50 µL streptavidin magnetic beads (Thermo Fisher Scientific), and then immersed with 300 µL buffer twice. An antagonist miR-506-3p probe was regarded as a NC. Trizol was utilized for extracting total RNA to measure miR-506-3p expression by RT-qPCR.

### Dual luciferase reporter gene assay

Kcnq1ot1 and SYPL1 3'UTR sequences containing miR-506-3p binding site or mutant binding site were synthesized by Generalbio (Anhui, China) to produce Kcnq1ot1-wilde type (WT) and SYPL1-WT or Kcnq1ot1-mutant (MUT) and SYPL1-MUT. These reporter vectors were co-transfected into Cal-27 cells with miR-506-3p mimic or mimic NC through Lipofectamine 3000 (Invitrogen) for determining luciferase activities on a luciferase reporter gene kit (Beyotime) [23].

### Subcutaneous xenograft nude mice model

The nude mice (4–5 weeks old) were kept in a specific pathologic-free environment. To establish the subcutaneous xenograft model, nude mice were injected subcutaneously with Cal-27 or SCC25 cells stably expressing si-Kcnq1ot1 or si-NC (5 mice per group). Since that, tumor growth was monitored every 5 d when the tumor masses were identified, and tumor volume was estimated by (length × width^2^) × 0.52. Mice were euthanized a month later, and the tumors were subsequently excised, photographed, with the tissues sections selected for further experiments [17].

### Transferase-mediated deoxyuridine triphosphate-biotin nick end labeling (TUNEL) staining

Tumor tissues were fixed with 10% paraformaldehyde and embedded in paraffin. Then, tissues were interacted with the TUNEL mixture (POD, Roche) and observed under a fluorescence microscope (Olympus) to calculate the positive rate.

### Statistical analysis

Data evaluation was completed on SPSS21.0 (IBM, NY, USA) and GraphPad Prism version 6.0 (GraphPad Software, San Diego). Measurement data were presented in mean ± standard deviation. The t test was employed to compare data between two groups. One-way analysis of variance (ANOVA) was indicated to evaluate data among multiple groups. Pearson test was indicated to correlation analysis. Log-rank test and Kaplan-Meier (K-M) curve were used to analyze the survival differences between the patients. Statistical significance was established with *P* < 0.05.

## Results

### Kcnq1ot1 is overexpressed in OSCC; silenced Kcnq1ot1 delays OSCC progression

**Fig. 1:**
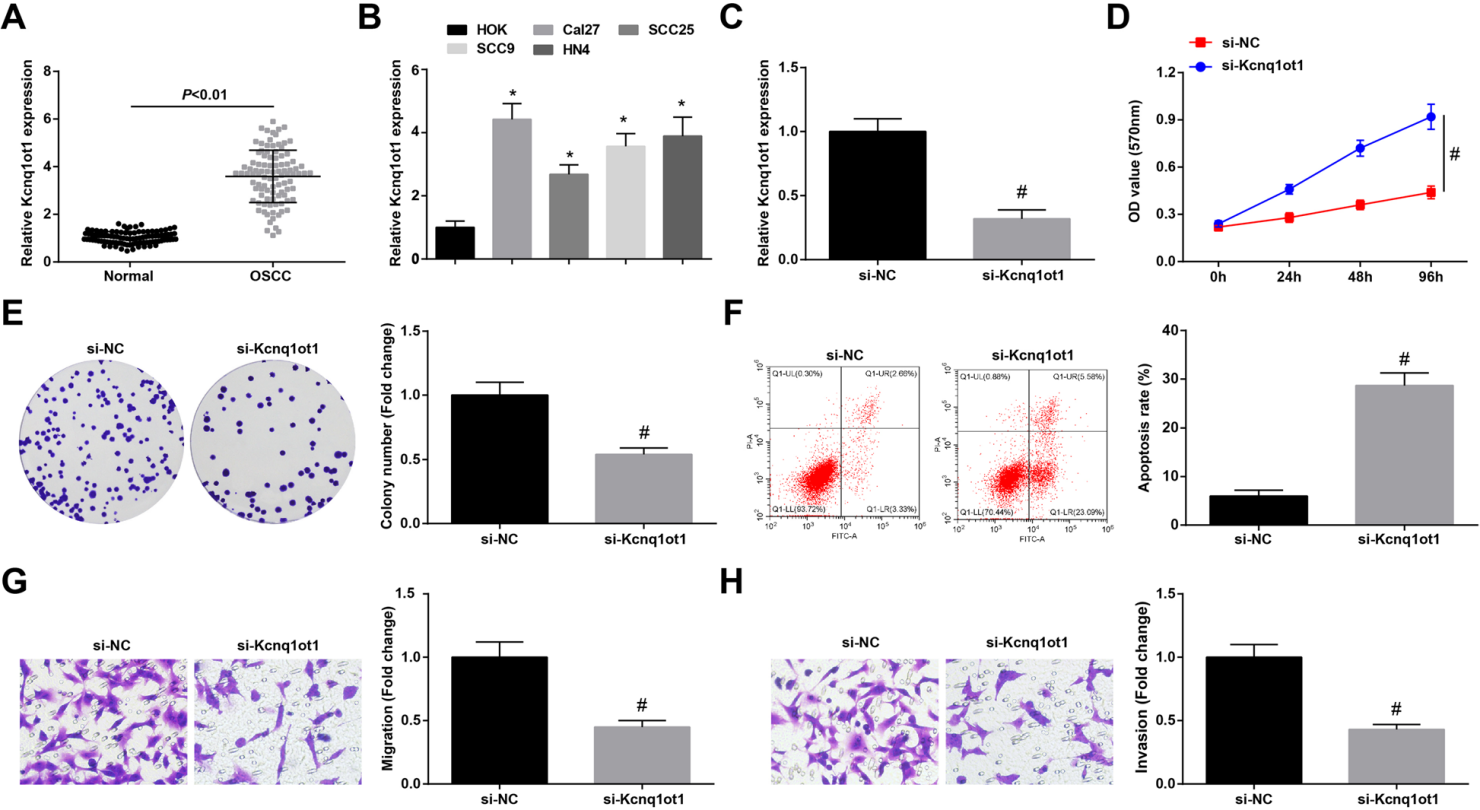
Kcnq1ot1 is overexpressed in OSCC; silenced Kcnq1ot1 delays Cal27 cell progression. **A** RT-qPCR for detecting Kcnq1ot1 expression in OSCC tissues and normal tissues, *n* = 98; **B** RT-qPCR for determining Kcnq1ot1 expression in OSCC cell lines and HOK cell line; **C** RT-qPCR for testing Kcnq1ot1 expression after down-regulating Kcnq1ot1; **D-E** MTT assay and colony formation assay for detecting cell proliferation after down-regulating Kcnq1ot1; **F** Flow cytometry for measuring cell apoptosis after down-regulating Kcnq1ot1; **G-H** Transwell assay for testing cell migration and invasion after down-regulating Kcnq1ot1; * *P* < 0.05 compared with HOK cells; # *P* < 0.05 compared with the si-NC group; Measurement data were expressed as mean ± standard deviation

Kcnq1ot1 has been proved to promote TSCC cells to proliferate [10], and it is up-regulated in tongue cancer [8]. Kcnq1ot1 expression in OSCC tissues and normal tissues (*n* = 98) was tested by RT-qPCR, showing a raised trend in OSCC tissues (Fig. 1A). In addition, its expression showed the same trend in OSCC cell lines (Cal-27, SCC-25, SCC-9 and HN4) versus to HOK cell line (Fig. 1B).

Kcnq1ot1 knockdown was performed in Cal27 (with highest Kcnq1ot1 expression compared with HOK cells) and in SCC25 cells (with lowest Kcnq1ot1 expression compared with HOK cells) to further survey its functional action in OSCC. Firstly, RT-qPCR proved that Kcnq1ot1 expression was effectively inhibited in Cal-27 and SCC25 cells (Fig. 1C; Supplementary Fig. 1A). Then, when detecting Cal-27 and SCC25 cell progression, the result data presented that cell viability, colony-forming ability, migration and invasion were all depressed, and apoptosis was induced upon downregulating Kcnq1ot1 (Fig. 1D-H; Supplementary Fig. 1B-F). The results hinted the repressive effects of silenced Kcnq1ot1 on OSCC cells.

### YY1 promotes Kcnq1ot1 transcription and elevates Kcnq1ot1 expression to promote OSCC progression

**Fig. 2:**
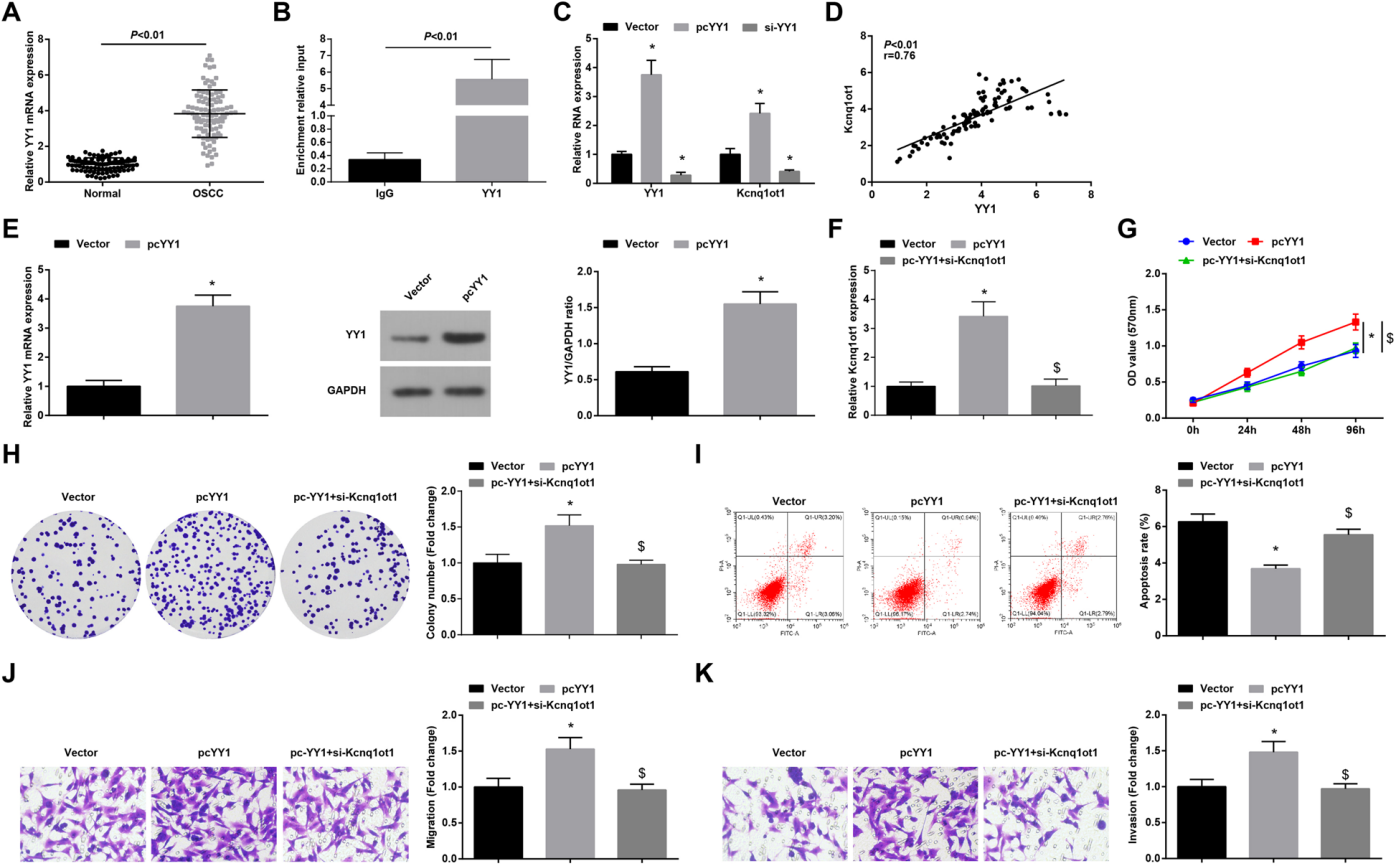
YY1 promotes Kcnq1ot1 transcription and elevates Kcnq1ot1 expression to promote OSCC progression. **A** RT-qPCR was performed to detect YY1 expression in OSCC tissues and normal tissues, *n* = 98; **B** ChIP assay was implemented to verify the binding relation between Kcnq1ot1 and YY1; **C** RT-qPCR was utilized to determine YY1 and Kcnq1ot1 expression after down-regulating or up-regulating YY1; **D** Pearson test was carried out to analyze the correlation between Kcnq1ot1 and YY1 expression in OSCC tissues, *n* = 98; E. RT-qPCR and Western blot assay were employed to test YY1 expression in cells in response to cotransfection of pc-YY1 and si-Kcnq1ot1; **F** RT-qPCR for detecting Kcnq1ot1 expression in cells in response to cotransfection of pc-YY1 and si-Kcnq1ot1; **G-H** MTT assay and colony formation assay were utilized to determine cell proliferation in cells in response to cotransfection of pc-YY1 and si-Kcnq1ot1; **I** Flow cytometry for measuring cell apoptosis in cells in response to cotransfection of pc-YY1 and si-Kcnq1ot1; **J-K** Transwell assay was carried out to detect cell migration and invasion in cells in response to cotransfection of pc-YY1 and si-Kcnq1ot1; * *P* < 0.05 compared with the Vector group; $ *P* < 0.05 compared with pcYY1 group; Measurement data were expressed as mean ± standard deviation

It has been confirmed that the abnormally expressed lncRNA is stimulated by transcriptional activation, which might be affected by transcription factors [24]. Meanwhile, YY1 activated LINC00673 to reinforce breast cancer cell proliferation through the miR-515-5p/microtubule affinity regulating kinase/Hippo signaling pathway [22]. YY1 expression was firstly examined in OSCC tissues and normal tissues and the outcome displayed that YY1 was overexpressed in OSCC tissues (Fig. 2A). To proceed, whether YY1 regulated Kcnq1ot1 in OSCC was explored via ChIP assay. The results reported that anti-YY1 pulled down Kcnq1ot1 promoter (Fig. 2B). Moreover, in Cal-27 cells expressing up-regulated YY1, Kcnq1ot1 expression was raised, and vice versa (Fig. 2C). Also, in OSCC tissues, there was a positive correlation between Kcnq1ot1 and YY1 expression (Fig. 2D).

Subsequently, the mechanism that YY1 mediated Kcnq1ot1 in OSCC was investigated. It was revealed that transfection with pc-YY1 could increase YY1 and Kcnq1ot1 expression in Cal27 cells, further transfection with si-Kcnq1ot1 could reduced Kcnq1ot1 expression (Fig. 2E, F).

Through in vitro experiments, it was measured that overexpressed YY1-induced enhancements of Cal-27 cell viability, colony-forming, invasion and migration abilities and impairment of apoptosis were offset by knockdown of Kcnq1ot1 (Fig. 2G-K). In short, YY1 promoted Kcnq1ot1 transcription and up-regulated its expression, thereby promoting OSCC development.

### Knocking down Kcnq1ot1 suppresses tumorigenic ability of OSCC cells in mice

**Fig. 3:**
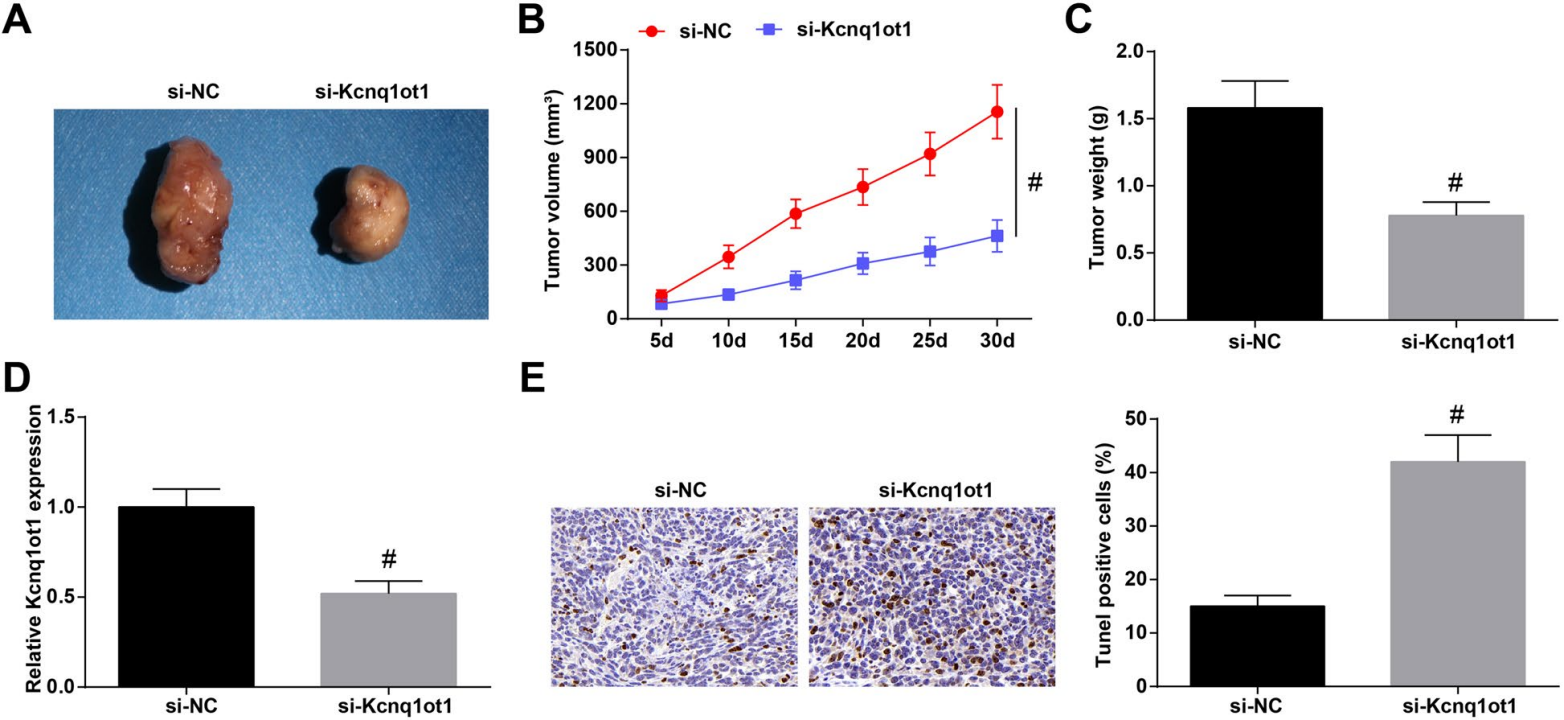
Knocking down Kcnq1ot1 suppresses tumorigenic ability of Cal27 cells in mice. **A** Xenografted tumors obtained after 30 days of tumorigenesis; **B** Tumor growth curve in mice; **C** Tumor weight in miec; **D** RT-qPCR for the detection of Kcnq1ot1 expression in tumors; E. TUNEL staining for analyzing the tumor cell apoptosis. # *P* < 0.05 compared with the si-NC group. Measurement data were expressed as mean ± standard deviation

As to the issue that how Kcnq1ot1 acted in OSCC in vivo, Cal-27 cells stably expressing sh-Kcnq1ot1 or sh-NC were injected into mice. The tumors expressing with sh-Kcnq1ot1 showed reduced volume and weight (Fig. 3A-C), decreased Kcnq1ot1 expression (Fig. 3D) and enhanced cell apoptosis (Fig. 3E). Taken together, knockdown of Kcnq1ot1 suppressed OSCC tumor growth.

**Fig. 4:**
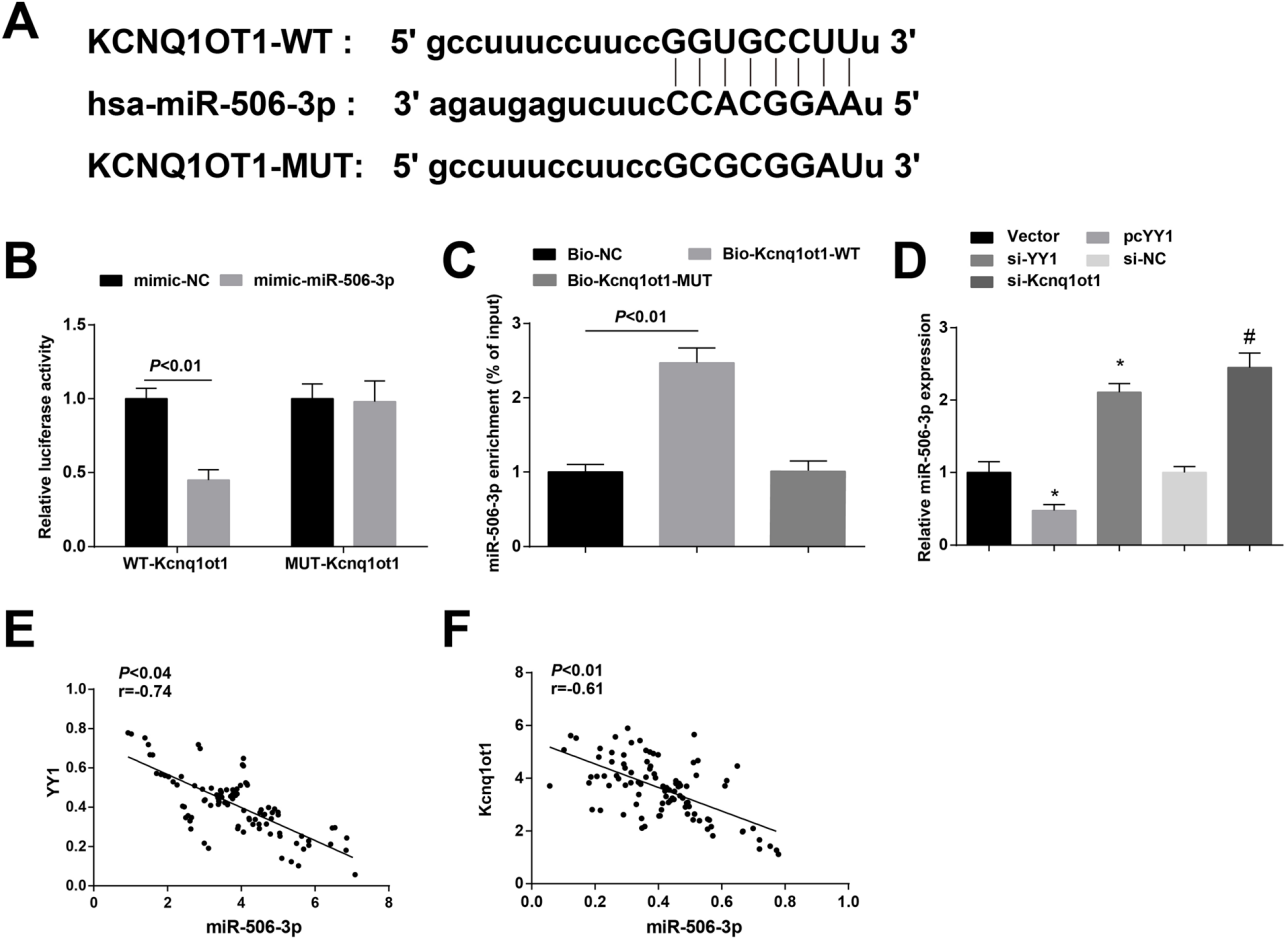
Kcnq1ot1 competitively binds to miR-506-3p. **A** The website predicted the targeting relationship between Kcnq1ot1 and miR-506-3p; **B-C** Dual luciferase reporter gene assay and RNA pull down assay were conducted to verify the targeting relationship between Kcnq1ot1 and miR-506-3p; **D** RT-qPCR for determining miR-506-3p expression in cells downregulating YY1 or Kcnq1ot1; **E** Pearson test was carried out to analyze the correlation between miR-506-3p and YY1 in OSCC tissues, *n* = 98; **F** Pearson test was implemented to analyze the correlation between miR-506-3p and Kcnq1ot1 in OSCC tissues. * *P* < 0.05 compared with the Vector group; # *P* < 0.05 compared with the si-NC group; Measurement data were expressed as mean ± standard deviation

miR-506-3p was a potential therapeutic target in cancer cells, as evidenced by its suppressive effects on nasopharyngeal carcinoma, osteosarcoma and gliomas [25–27]. In addition to protein-coding genes, miRNAs can target lncRNAs. Among candidate targets of miR-506-3p predicted by the starBase website, we found the binding sequences between miR-506-3p and Kcnq1ot1 (Fig. 4A). Furthermore, dual luciferase reporter gene assay revealed that miR-506-3p mimic had impaired effects on the luciferase activity of WT-Kcnq1ot1 but not on that of MUT-Kcnq1ot1 in Cal-27 cells (Fig. 4B). RNA pull down assay detected that in Cal-27 cells, miR-506-3p was enriched in the Bio-Kcnq1ot1-WT group but not in the Bio-Kcnq1ot1-MUT group (Fig. 4C). RT-qPCR outlined that miR-506-3p expression was heightened upon YY1 or Kcnq1ot1 knockdown (Fig. 4D). YY1 and Kcnq1ot1 expression in OSCC tissues were found to be negatively correlated with miR-506-3p expression (Fig. 4E, F). It could be summarized that YY1-mediated Kcnq1ot1 competitively bound to miR-506-3p to regulate miR-506-3p expression.

### Elevating miR-506-3p restrains tumorigenesis of OSCC in mice

**Fig. 5:**
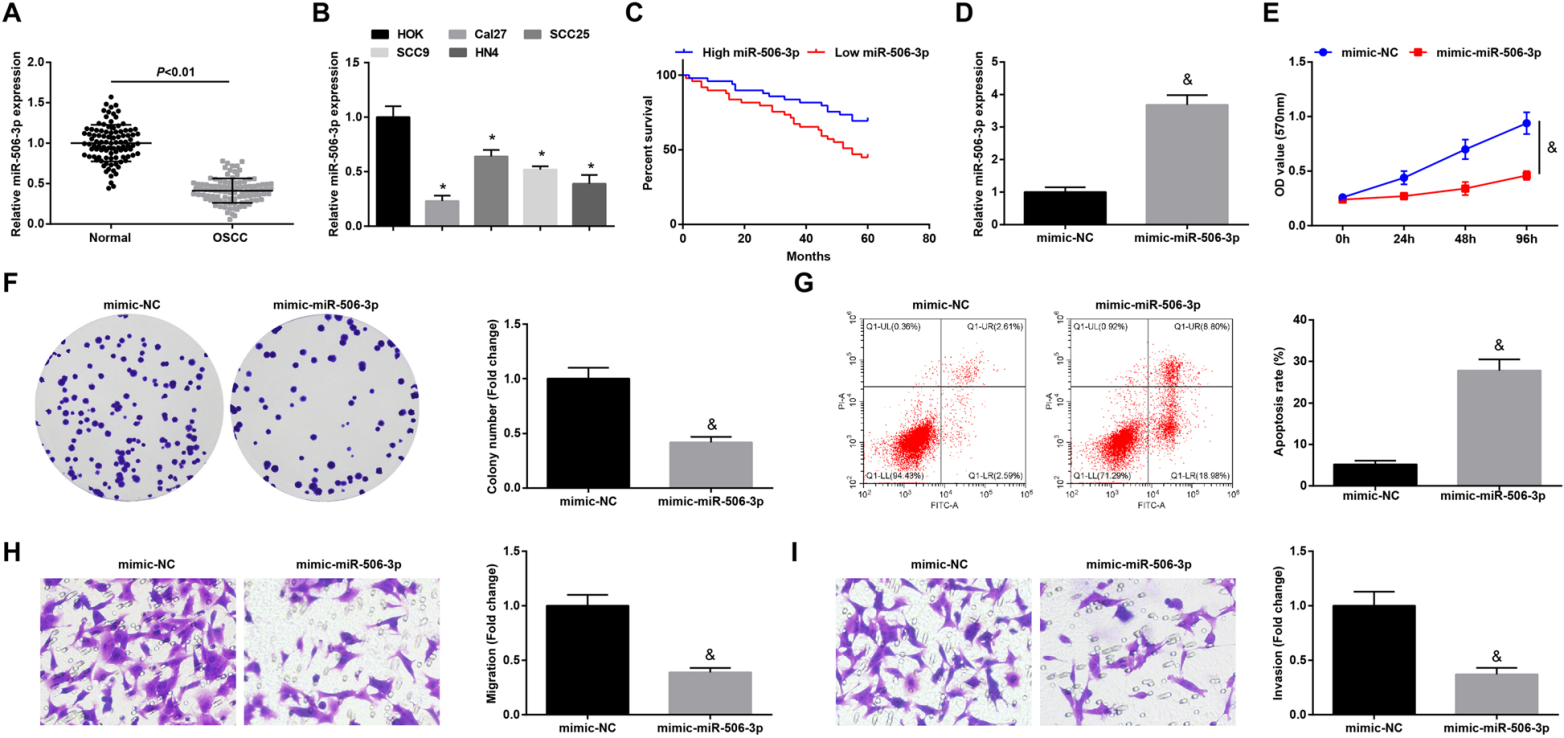
Elevating miR-506-3p restrainsCal27 cell progression. **A** RT-qPCR for the determination of miR-506-3p expression in OSCC tissues and normal tissues, *n* = 98; **B** RT-qPCR for testing miR-506-3p expression in OSCC cell lines and HOK cell line; **C** Correlation between miR-506-3p expression and survival prognosis of OSCC patients; **D** RT-qPCR was performed to measure miR-506-3p expression after up-regulating miR-506-3p; **E–F** MTT assay and colony formation assay were conducted to measure cell proliferation after up-regulating miR-506-3p; **G** Flow cytometry for the determination of cell apoptosis after up-regulating miR-506-3p; **H-I** Transwell assay for the detection of cell migration and invasion after up-regulating miR-506-3p; * *P* < 0.05 compared with HOK cells; & *P* < 0.05 compared with the mimic-NC group; Measurement data were expressed as mean ± standard deviation

RT-qPCR examined miR-506-3p expression in clinical tissues in OSCC. Revealed by the experimental results, miR-506-3p expression was suppressed in OSCC tissues (Fig. 5A). Consistently, miR-506-3p expression in OSCC cells was lower than that in HOK cells (Fig. 5B). Through the 5-year prognosis statistics of patients with OSCC, it was found that low expression of miR-506-3p was related to a poor prognosis of patients (Fig. 5C). In addition, miR-506-3p expression was related to the tumor clinical stage and lymph node metastasis, but not to gender, age, and tumor differentiation (Supplementary Table 2).

Up-regulated miR-506-3p was introduced into Cal-27 cells to further clarify its function in OSCC (Fig. 5D). The findings highlighted that restored miR-506-3p impaired OSCC cell progression (Fig. 5E-I). We performed the same experiments in SCC25 cells, and the results showed that the trend was consistent with Cal27 cells (Supplementary Fig. 2A-F).

### Kcnq1ot1 regulates miR-506-3p to mediate SYPL1 expression

**Fig. 6:**
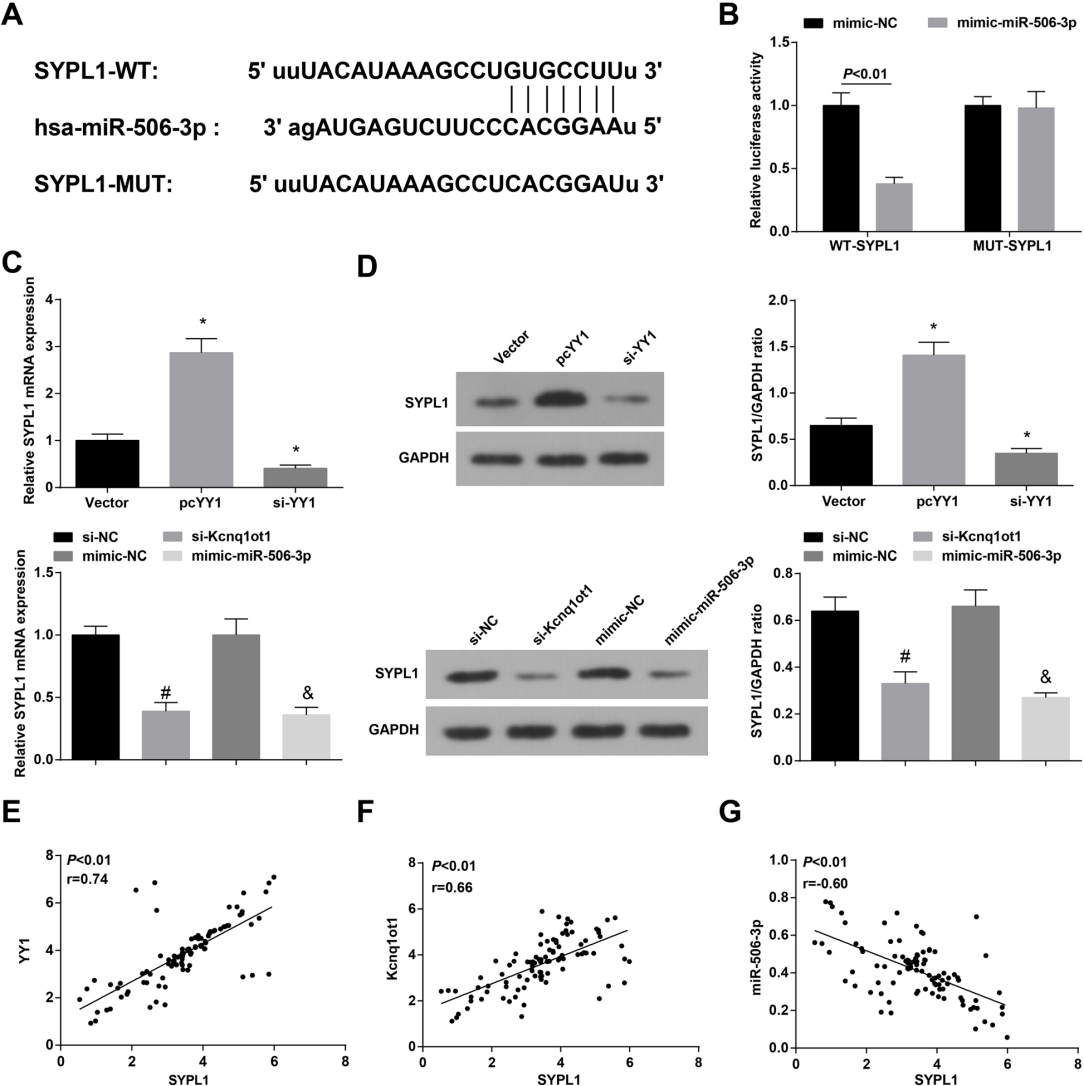
Kcnq1ot1 regulates miR-506-3p to mediate SYPL1. **A** starBase website predicted the targeting relationship between miR-506-3p and SYPL1; **B** Dual luciferase reporter gene assay was utilized to confirm the targeting relationship between miR-506-3p and SYPL1; **C-D** RT-qPCR and western blot assay were conducted to detect SYPL1 expression; **E–G** Pearson test was employed to analyze the correlation between SYPL1 and YY1/Kcnq1ot1/miR-506-3p in OSCC tissues; * *P* < 0.05 compared with the Vector group; # *P* < 0.05 compared with the si-NC group; & *P* < 0.05 compared with mimic-NC group; Measurement data were expressed as mean ± standard deviation

miR-506-3p could regulate target genes in cancer cells [27]. It was assumed that miR-506-3p repressed OSCC progression by binding to its target gene. SYPL1, a member of SYP family of proteins, is originally reported to be a protein associated with neuroendocrine-specific synaptophysin, and recently discovered in non-nerve tissues [28]. SYPL1 was predicted as the target gene of miR-506-3p to participate in OSCC. On the bioinformatics website, it was disclosed that SYPL1 may be a potential target of miR-506-3p (Fig. 6A). Next, dual luciferase report gene assay validated (Fig. 6B) that miR-506-3p mimic undermined the luciferase activity of SYPL1-WT, evidencing the targeting relation between these two.

### Kcnq1ot1 competitively binds to miR-506-3p

To delineate the regulatory mechanism among Kcnq1ot1, SYPL1 and miR-146a-5p, si-YY1, si-Kcnq1ot1 and mimic-miR-506-3p was transfected in Cal-27 cells, respectively. The data showed that SYPL1 expression was always repressed after transfection with the above plasmids. Transfection with pc-YY1 could augment SYPL1 expression in cells (Fig. 6C, D). In OSCC tissues, SYPL1 expression was positively correlated with YY1 and Kcnq1ot1 expression, and negatively with miR-506-3p expression (Fig. 6E-G).

### Inhibiting SYPL1 restricts OSCC progression

**Fig. 7:**
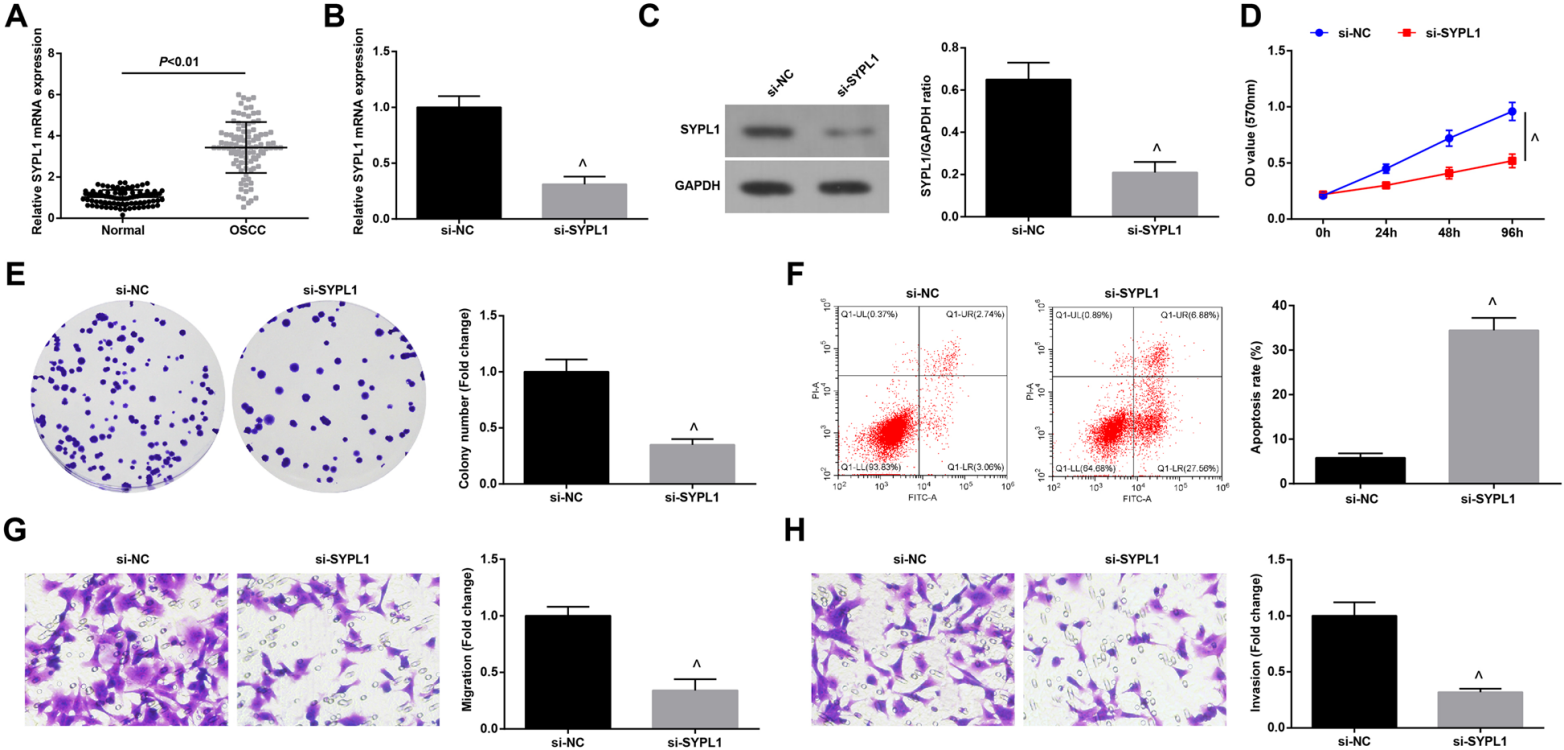
Inhibiting SYPL1 restricts Cal27 cell progression. **A** RT-qPCR was performed to test SYPL1 expression in OSCC tissues and normal tissues, *n* = 98; **B-C** RT-qPCR and Western blot assay were conducted to determine SYPL1 expression after down-regulating SYPL1; **D-E**. MTT assay and colony formation assay were carried out to detect cell proliferation after down-regulating SYPL1; **F** Flow cytometry was utilized to measure cell apoptosis after down-regulating SYPL1; **G-H** Transwell assay was carried out to detect cell migration and invasion after down-regulating SYPL1; ^ *P* < 0.05 compared with the si-NC group; Measurement data were expressed as mean ± standard deviation

Overexpressed SYPL1 was closely tied up with malignant clinicopathological features in hepatocellular carcinoma [28]. To explore the effects of SYPL1 on OSCC, its expression in OSCC tissues and normal tissues was determined by RT-qPCR. The results demonstrated that SYPL1 expression was augmented in OSCC tissues (Fig. 7A).

Transfection with si-SYPL1 was conducted in Cal-27 cells to down-regulate SYPL1 expression in OSCC (Fig. 7B, C). As a result of SYPL1 inhibition, the malignant activities of OSCC cells were disrupted (Fig. 7D-H). We performed the same experiments in SCC25 cells and found that inhibiting SYPL1 repressed the growth of SCC25 cells (Supplementary Fig. 3A-F).

## Discussion

OSCC is defined as one of the tumors in the oral epithelium [29]. Abnormalities in YY1, Kcnq1ot1, miR-506-3p, and SYPL1 have been manifested in cancer progression. Given that, it is speculated that targeting YY1/Kcnq1ot1/miR-506-3p/SYPL1 axis may possess the potential to treat OSCC. For verifying the speculation, this research was launched to obtain the major result that YY1-mediated Kcnq1ot1 depresses miR-506-3p to up-regulate SYPL1, thereafter to facilitate OSCC development.

To start, YY1 and Kcnq1ot1 expression in OSCC was determined, along with their interactions in OSCC cell progression. The experimental results presented that YY1 and Kcnq1ot1 were both overexpressed in OSCC, and YY1 activated Kcnq1ot1 transcription to elevate Kcnq1ot1 expression. Functionally evidenced by various assays, YY1 or Kcnq1ot1 overexpression gave the opportunity for OSCC cells to act offensively while Kcnq1ot1 knockdown disrupted the cellular fate. Moreover, Kcnq1ot1 knockdown performed in vivo also exhibited the same inhibitory effects on tumorgentic ability of OSCC cells in vivo. Impressively, a novel study has mentioned that YY1 expression is raised in oral cancer, facilitating proliferation, angiogenesis and metastasis of oral cancer cells [30]. Moreover, another article has reported YY1 knockdown sensitizes TSCC cells to ionizing radiation [6]. In head and neck squamous cell carcinoma, YY1 expression goes toward an up-regulation while YY1 depletion strengthens cell apoptosis and interferes cell proliferation, migration and invasion [31]. Furthermore, overexpressed YY1 in ESCC has been revealed to promote ESCC cell invasion and migration, as well as metastatic behaviors [32, 33]. It is academically reflected that transcriptional activation of Kcnq1ot1 is stimulated by YY1 [7]. Cisplatin-resistant tongue cancer tissues and cells witness the enhancement of Kcnq1ot1 and its silencing is attributable to restricted pro-proliferative, pro-migrating, pro-invasive properties of malignant cells [8]. Specifically, Kcnq1ot1 expression reaches a high level in OSCC tissues and cells and depleting it muffles invasion and induces apoptosis of OSCC cells [9]. Observed in another work, incremental Kcnq1ot is the activator for TSCC proliferation while reduced Kcnq1ot functions reversely [10].

Next, miR-506-3p was predicted and verified to interact with Kcnq1ot1. As manifested, miR-506-3p was down-regulated in OSCC. By up-regulating miR-506-3p, the biological behaviors of OSCC cells were disturbed. An existed literature has shown Kcnq1ot1 targeting miR-506-3p [11]. miR-506 expression is apt to decline in laryngeal squamous cell carcinoma and restored miR-506 curbs cell proliferation, migration and invasion [34]. Besides that, in esophageal cancer, miR-506 also shows a reduced level while its enforced expression destroys cell proliferation [35]. In light of its presentation in OSCC, miR-506 is down-regulated, whereas miR-506 up-regulation depresses proliferation, migration and invasion of OSCC cells [14]. Previously discussed, inhibited miR-506-3p expression is noticeable in ovarian cancer and prostate cancer, and accumulated miR-506-3p is conducive in undermining cell viability and triggering apoptosis [36, 37].

Subsequently, SYPL1 was validated to be targeted by miR-506-3p, and its expression was negatively connected with miR-506-3p expression but positively with YY1 and Kcnq1ot1 expression. Followed by that, SYPL1 down-regulation in OSCC cells was found to obstruct cells to behave aggressively. The targeting connection between miR-506-3p and SYPL1 has been seldom identified previously. Clinically, SYPL1 expression is characterized with an increase in hepatocellular carcinoma, unfortunately resulting in miserable overall survival and disease-free survival [28]. Consistent the study finding in the present work, another research focused on CRC has reported the overexpressed SYPL1 [15]. From all of the listed reports, it is easy to abstract that the manifestations of these four factors (YY1, Kcnq1ot1, miR-506-3p and SYPL1) in the malignant phenotypes of cancers are consonant with the findings in the present work.

## Conclusion

To sum up, it is delineated in this article that YY1-mediated Kcnq1ot1/miR-506-3p/SYPL1 axis promotes OSCC cell progression. Much scientific and scrutinized studies are at wanting for further confirmation and extension of the obtained results in a larger cohort.

## Supplementary Information

**Additional file 1: Supplementary Figure 1.** Downregulation of Kcnq1ot1 inhibits the progression of SCC25 cells. A. RT-qPCR for detecting Kcnq1ot1 expression after down-regulating Kcnq1ot1; B-C. MTT assay and colony formation assay for determining cell proliferation after down-regulating Kcnq1ot1; D. Flow cytometry for measuring cell apoptosis after down-regulating Kcnq1ot1; E-F. Transwell assay for testing cell migration and invasion after down-regulating Kcnq1ot1; * P < 0.05 compared with the si-NC group; Measurement data were expressed as mean ± standard deviation.

**Additional file 2: Supplementary Figure 2.** Elevating miR-506-3p restrains the functions of SCC25 cells. A. RT-qPCR for determining miR-506-3p expression after up-regulating miR-506-3p; B-C. MTT assay and and colony formation assay for testing cell proliferation after up-regulating miR-506-3p; D. Flow cytometry for measuring cell apoptosis after up-regulating miR-506-3p; E-F. Transwell assay for the determination of cell migration and invasion after up-regulating miR-506-3p; * P < 0.05 compared with the mimic-NC group; Measurement data were expressed as mean ± standard deviation.

**Additional file 3: Supplementary Figure 3.** Inhibiting SYPL1 restricts the development of SCC25 cells. A. RT-qPCR fordetecting SYPL1 expression after down-regulating SYPL1; B-C. MTT assay and colony formation assay for determining cell proliferation after down-regulating SYPL1; D. Flow cytometry for analyzing cell apoptosis after down-regulating SYPL1; E-F. Transwell assay for testing cell migration and invasion after down-regulating SYPL1; * P < 0.05 compared with the si-NC group; Measurement data were expressed as mean ± standard deviation.

**Additional file 4: Supplementary Table 1.** Primer sequences for genes in this study. **Supplementary Table 2.** The correlation between miR-506-3p expression and clinicopathological characteristics of patients with oral squamous cell carcinoma.

## Data Availability

The original contributions presented in the study are included in the article/Supplementary Material, further inquiries can be directed to the corresponding author.
