# Supplementary figures and images for: YY1 accelerates oral squamous cell carcinoma progression through long non-coding RNA Kcnq1ot1/microRNA-506-3p/SYPL1 axis
Source: J Ovarian Res. 2022 Jul 1;15:77. doi: 10.1186/s13048-022-01000-5 (PMC9250217; doi:10.1186/s13048-022-01000-5)

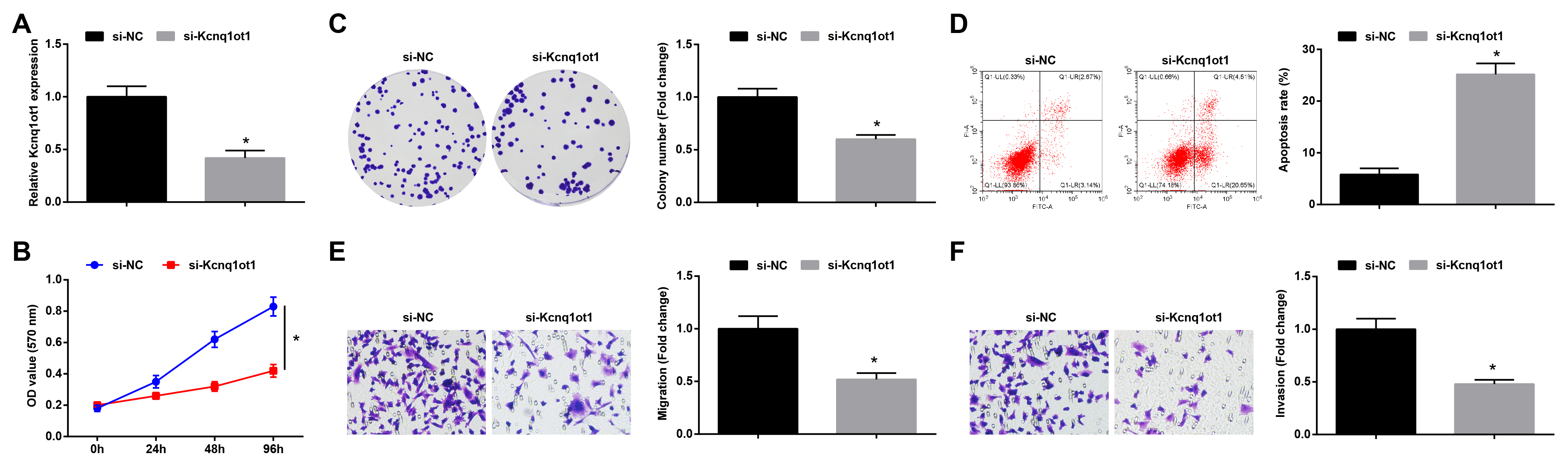

Supplement: Supplementary file 1 — Additional file 1: Supplementary Figure 1. Downregulation of Kcnq1ot1 inhibits the progression of SCC25 cells. A. RT-qPCR for detecting Kcnq1ot1 expression after down-regulating Kcnq1ot1; B-C. MTT assay and colony formation assay for determining cell proliferation after down-regulating Kcnq1ot1; D. Flow cytometry for measuring cell apoptosis after down-regulating Kcnq1ot1; E-F. Transwell assay for testing cell migration and invasion after down-regulating Kcnq1ot1; * P < 0.05 compared with the si-NC group; Measurement data were expressed as mean ± standard deviation. [file 13048_2022_1000_MOESM1_ESM.jpg]

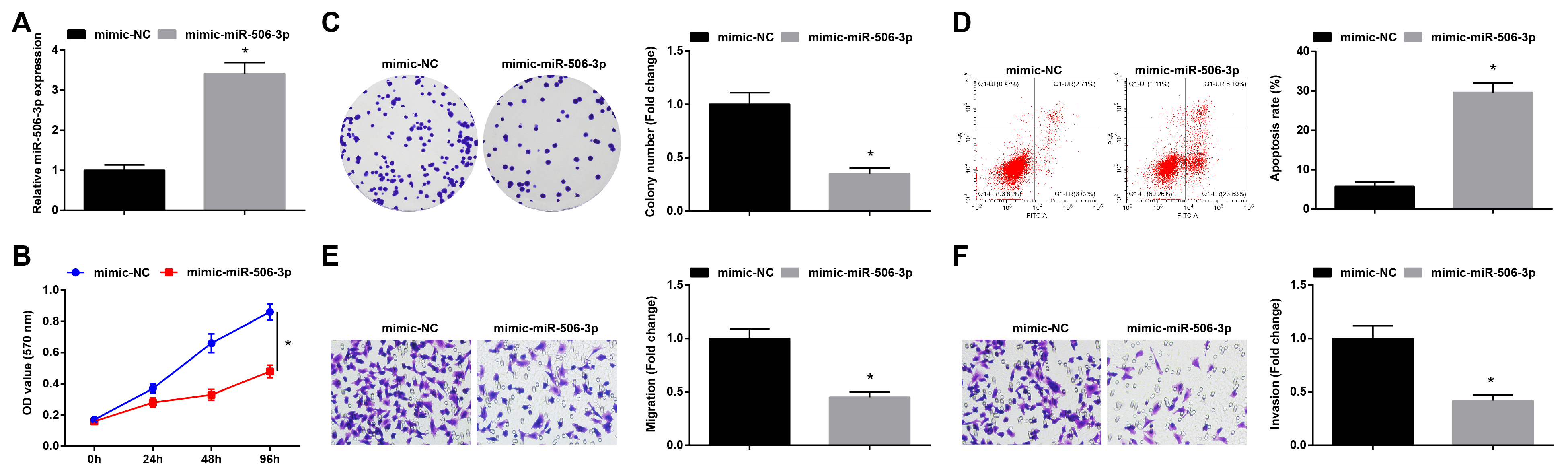

Supplement: Supplementary file 2 — Additional file 2: Supplementary Figure 2. Elevating miR-506-3p restrains the functions of SCC25 cells. A. RT-qPCR for determining miR-506-3p expression after up-regulating miR-506-3p; B-C. MTT assay and and colony formation assay for testing cell proliferation after up-regulating miR-506-3p; D. Flow cytometry for measuring cell apoptosis after up-regulating miR-506-3p; E-F. Transwell assay for the determination of cell migration and invasion after up-regulating miR-506-3p; * P < 0.05 compared with the mimic-NC group; Measurement data were expressed as mean ± standard deviation. [file 13048_2022_1000_MOESM2_ESM.jpg]

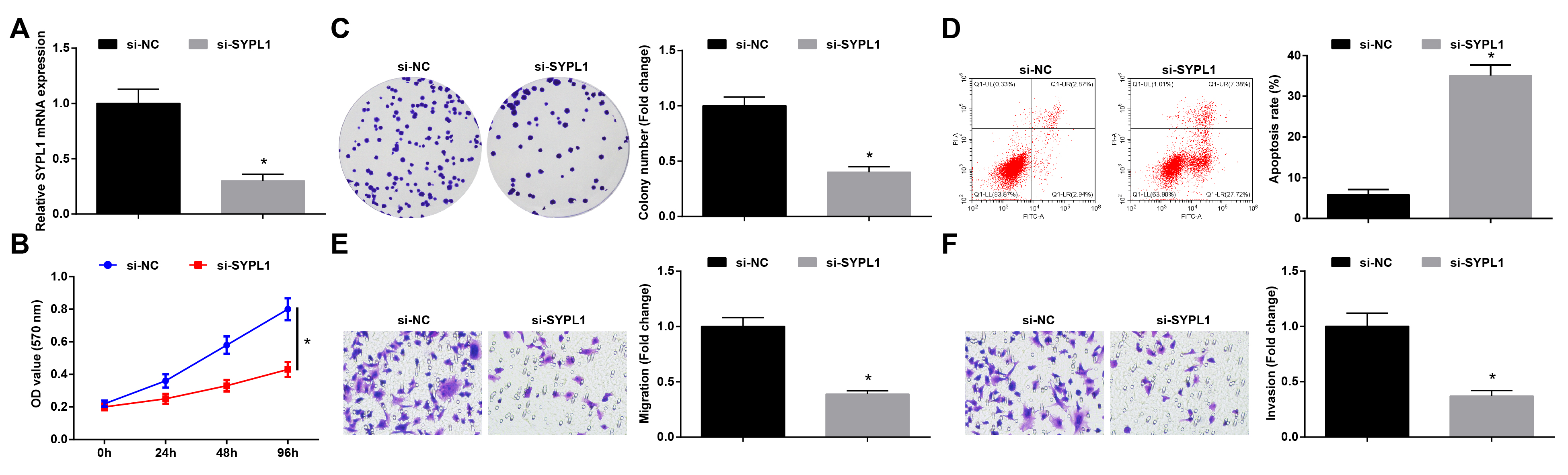

Supplement: Supplementary file 3 — Additional file 3: Supplementary Figure 3. Inhibiting SYPL1 restricts the development of SCC25 cells. A. RT-qPCR fordetecting SYPL1 expression after down-regulating SYPL1; B-C. MTT assay and colony formation assay for determining cell proliferation after down-regulating SYPL1; D. Flow cytometry for analyzing cell apoptosis after down-regulating SYPL1; E-F. Transwell assay for testing cell migration and invasion after down-regulating SYPL1; * P < 0.05 compared with the si-NC group; Measurement data were expressed as mean ± standard deviation. [file 13048_2022_1000_MOESM3_ESM.jpg]
