# Supplementary material for: YY1 accelerates oral squamous cell carcinoma progression through long non-coding RNA Kcnq1ot1/microRNA-506-3p/SYPL1 axis
Source: J Ovarian Res. 2022 Jul 1;15:77. doi: 10.1186/s13048-022-01000-5 (PMC9250217; doi:10.1186/s13048-022-01000-5)
Supplement: Supplementary file 4 — Additional file 4: Supplementary Table 1. Primer sequences for genes in this study. Supplementary Table 2. The correlation between miR-506-3p expression and clinicopathological characteristics of patients with oral squamous cell carcinoma. [file 13048_2022_1000_MOESM4_ESM.docx]

**Supplementary Table 1** Primer sequences for gens in this study

| Primer sequences | Forward (5’→3’) | Reverse (5’→3’) |
| --- | --- | --- |
| YY1 | AAGAGCGGCAAGAAGAGTTAC | CAACCACTGTCTCATGGTCAATA |
| Kcnq1ot1 | AGGGTGACAGTGTTTCATAGGCT | GAGGCACATTCATTCGTTGGT |
| miR-506-3p | TAAGGCACCCTTCTGAGTAGA | Universal primer |
| SYPL1 | TATGTTGGCTACACGAGTCTGT | ACAAGGCGGAAGTTCATCAATAA |
| U6 | GCTTCGGCAGCACATATACTAAAAT | CGCTTCACGAATTTGCGTGTCAT |
| GAPDH | AAGAAGGTGGTGAAGCAGGC | GTCAAAGGTGGAGGAGTGGG |

Note: YY1, Ying Yang1; Kcnq1ot1, long non-coding RNA KCNQ1 overlapping transcript 1; miR-506-3p, microRNA-506-3p; SYPL1, synaptophysin like 1; GAPDH, glyceraldehyde-3-phosphate dehydrogenase

**Supplementary Table 2** The correlation between miR-506-3p expression and clinicopathological characteristics of patients with oral squamous cell carcinoma

| Clinicopathological characteristics | NO. | miR-506-3p expression | | *P* |
| --- | --- | --- | --- | --- |
|  |  | High | Low |  |
| Gender |  |  |  | 0.304 |
| Male | 40 | 17 | 23 |  |
| Female | 58 | 32 | 26 |  |
| Age (Years) |  |  |  | 0.225 |
| < 50 | 47 | 20 | 27 |  |
| ≥ 50 | 51 | 29 | 22 |  |
| Clinical stage |  |  |  | 0.019 |
| I/II | 34 | 23 | 11 |  |
| III/Ⅳ | 64 | 26 | 38 |  |
| Differentiation |  |  |  | 0.011 |
| Well/moderate | 72 | 42 | 30 |  |
| Poor | 26 | 7 | 19 |  |
| Lymph node metastasis |  |  |  | 0.015 |
| Yes | 49 | 18 | 31 |  |
| No | 49 | 31 | 18 |  |
